# In vitro assessment of *Pediococcus acidilactici* Kp10 for its potential use in the food industry

**DOI:** 10.1186/s12866-017-1000-z

**Published:** 2017-05-23

**Authors:** Sahar Abbasiliasi, Joo Shun Tan, Fatemeh Bashokouh, Tengku Azmi Tengku Ibrahim, Shuhaimi Mustafa, Faezeh Vakhshiteh, Subhashini Sivasamboo, Arbakariya B. Ariff

**Affiliations:** 1 Department of Microbiology, Faculty of Biotechnology and Biomolecular Sciences, Universiti Putra Malaysia, 43400 UPM Serdang, Selangor Malaysia; 2 Bioprocessing and Biomanufacturing Research Centre, Faculty of Biotechnology and Biomolecular Sciences, Universiti Putra Malaysia, 43400 UPM Serdang, Selangor Malaysia; 3 School of Industrial Technology, Universiti Sains Malaysia, 11800 George Town, Penang Malaysia; 4 Institute of Bioscience, Universiti Putra Malaysia, 43300 Serdang, Selangor Malaysia; 5 Faculty of Veterinary Medicine, Universiti Putra Malaysia, 43400 UPM Serdang, Selangor Malaysia

**Keywords:** *Pediococcus acidilactici* Kp10, Probiotic, Starter culture, Adhesion property, Proteolytic, Food-borne pathogens, Food industry

## Abstract

**Background:**

Selection of a microbial strain for the incorporation into food products requires in vitro and in vivo evaluations. A bacteriocin-producing lactic acid bacterium (LAB), *Pediococcus acidilactici* Kp10, isolated from a traditional dried curd was assessed in vitro for its beneficial properties as a potential probiotic and starter culture. The inhibitory spectra of the bacterial strain against different gram-positive and gram-negative bacteria, its cell surface hydrophobicity and resistance to phenol, its haemolytic, amylolytic and proteolytic activities, ability to produce acid and coagulate milk together with its enzymatic characteristics and adhesion property were all evaluated in vitro.

**Results:**

*P. acidilactici* Kp10 was moderately tolerant to phenol and adhere to mammalian epithelial cells (Vero cells and ileal mucosal epithelium). The bacterium also exhibited antimicrobial activity against several gram-positive and gram-negative food-spoilage and food-borne pathogens such as *Listeria monocytgenes* ATCC 15313, *Salmonella enterica* ATCC 13311, *Shigella sonnei* ATCC 9290, *Klebsiella oxytoca* ATCC 13182, *Enterobacter cloaca* ATCC 35030 and *Streptococcus pyogenes* ATCC 12378. The absence of haemolytic activity and proteinase (trypsin) and the presence of a strong peptidase (leucine-arylamidase) and esterase-lipase (C4 and C8) were observed in this LAB strain. *P. acidilactici* Kp10 also produced acid, coagulated milk and has demonstrated proteolytic and amylolactic activities.

**Conclusion:**

The properties exhibited by *P. acidilactici* Kp10 suggested its potential application as probiotic and starter culture in the food industry.

## Background

The importance of proper selection of the bacterial strains for incorporation in food products is related to the considerable variations of the beneficial properties among different strains. Lactic acid bacteria (LAB) which are used worldwide have been focused in recent years for a variety of fermented foods production [1].

LAB play an important role in improving the nutritional and keeping qualities of foods by virtue of the organic acids produced during fermentation of the raw materials [2]. At the industrial scale, short fermentation duration is preferred in order to increase the plant output as well as to reduce microbial contamination. The use of LAB as a starter culture in food fermentation will increase the fermentation rates and also will improve product quality [3] due to LAB versatile metabolic characteristics such as acidification and proteolytic activities and ability to synthesize metabolites such as bacteriocin [4, 5]. Thus, the isolation and characterization of new strains of LAB for broader industrial applications is currently of industrial importance.

LAB species presence in traditional foods of Southeast Asian countries have not been extensively investigated and there is every likelihood that some species could be of commercial potential [1]. With the realization that there is a need to identify new strains with useful characteristics, in our previous study we had identified and characterized the LAB strain with ability to produce bacteriocin-like inhibitory substances (BLIS) for potential applications in the food industry. The isolate, *P. acidilactici* Kp10, could be a potential probiotic as it exerted beneficial and positive effects on the intestinal flora which included tolerance to bile salts (0.3%) and acidic conditions (pH 3), produced *β-*galactosidase, stable in a wide range of pH (2–9) and not resistant to vancomycin. Most interesting, the LAB strain showed the highest level of BLIS activity against *Listeria monocytogenes*, a virulent food pathogenic bacterium. To further substantiate its probiotic potential and application as a starter culture the present study further evaluated in vitro other physicochemical properties of *P. acidilactici* Kp10 which include inhibitory spectra of activities against different gram-positive and gram negative bacteria, cell surface hydrophobicity, resistance to phenol, haemolytic, amylolytic and proteolytic activities, ability to produce acid and coagulate milk and enzymatic characterization along with its adhesive properties.

## Methods

### Microorganism and maintenance

Isolation and characterization of the bacterium, *P. acidilactici*Kp10, used in this study were as described previously [1]. The culture was maintained on agar slopes at 4 °C and prior to its use in the present study the culture was sub-cultured twice in M17 broth (Merck, Darmstadt, Germany).

### Determination of probiotic properties

#### Inhibitory activity

The inhibitory activities of *P. acidilactici* Kp10 against different gram-positive and gram-negative bacteria (*Listeria monocytogenes* ATCC 15313, *Salmonella enterica* ATCC 13311, *Shigella sonnei* ATCC 9290, *Klebsiella oxytoca* ATCC 13182, *Enterobacter cloaca* ATCC 35030, *Streptococcus pyogenes* ATCC 12378) were determined according to the method as described in our previous study. Briefly, antimicrobial activity of *P. acidilactici* Kp10 was assessed by the agar well diffusion method using cell-free culture supernatants (CFCS). *P. acidilactici* Kp10 was grown in M17 broth at 30 °C for 24 h and the cultures were centrifuged at 12,000 g for 20 min at 4 °C (rotor model 1189, Universal 22R centrifuge, Hettich AG, Switzerland).

One hundred μL of the CFCS was placed into 6-mm wells of agar plates previously seeded with 1% (*v*/v) actively growing test strains. The plates were incubated at 37 °C for 24 h for the growth of test strains. After 24 h, the growth inhibition zones were measured, and the antimicrobial activity (AU mL^−1^) was calculated as described previously [6].

### Adhesion of *P. acidilactici* Kp10 on mammalian epithelial cells

#### Adhesion of *P. acidilactici* Kp10 to vero cells

Assessment of the adhesion of *P. acidilactici* Kp10 to Vero cells (African green monkey kidney cell line, ATCC CCL81) was performed by the method as described previously [7] with some modifications. Vero cells were cultured in Roswell Park Memorial Institute Medium (RPMI; Gibco, Grand Island, NY, USA) supplemented with 10% (*v*/v) fetal calf serum, 100 U/mL penicillin and 100 mg/mL streptomycin (Sigma, Switzerland). The cell lines were maintained in a humidified incubator (Binder, Tuttlingen, Germany) at 37 °C in atmosphere of 5% CO_2_ and 95% air. Cells with 80–85% confluence were washed three times with sterile phosphate-buffered saline (PBS: NaCl, 0·8, K_2_HPO_4_, 0·121, KH_2_PO_4_, 0·034, pH 7.2) and transferred (10^5^ cells/mL) onto cover slips placed in six-well plates containing fresh culture medium. The plates were incubated at 37 °C in an atmosphere of 5% CO_2_ and 95% air. Cell monolayers (10^5^ cells/mL) on glass cover slips were washed three times with PBS. Prior to the adhesion test, overnight culture of *P. acidilactic*i Kp10 was harvested and washed three times with PBS and centrifuged for 10 min at 3000×g. The bacterial cells (1 × 10^9^ CFU/mL in PBS) were resuspended in 1 mL of Dulbecco’s modified Eagle medium (DMEM) and transferred to the washed monolayer cells on cover slips, placed in six-well plates and incubated at 37 °C in an atmosphere of 5% CO_2_ and 95% air for 1 h.

For scanning electron microscopy (SEM) examination, the cells were fixed in 2.5% glutaraldehyde in 0.1 M sodium cacodylate buffer for 4–6 h and washed thrice in sodium cacodylate buffer. Samples were then postfixed in 1% aqueous osmium tetroxide, dehydrated in ascending grades of acetone concentrations (30, 50, 75, 80, 95 and 100%) critically point-dried and sputter coated with gold palladium.

#### Adhesion of *P. acidilactici* Kp10 to ileal mucosal epithelium

The method of Mäyrä-Mäukinen & Gyllenberg, [8] with slight modifications was employed to evaluate the adhesion of *P. acidilactici* Kp10 to ileal mucosal epithelium. Samples of goat ileum, obtained immediately after slaughter from a local abattoir were washed in PBS to remove the ingesta from the mucosal surface. The samples were transported back to the laboratory in cooled PBS and incubated in cell suspension of *P. acidilactici* Kp10 (10^9^ CFU/mL PBS) at 37 °C for 30 min. The samples were then prepared for scanning electron microscopy as described above.

#### Auto-aggregation and co-aggregation assays

The procedure as described by Polak-Berecka et al., [9] with some modifications was used to determine the specific cell–cell interactions using auto-aggregation and co-aggregation assays. Cells harvested at the stationary phase were collected by centrifugation (5000×g for 10 min at room temperature), washed twice and resuspended in PBS (pH 7.2). For both assays, the culture suspension was standardized to OD _600 nm_ = 1.0 (2 × 10^8^ CFU/mL). For auto-aggregation assay, 5 mL of bacterial suspension was vortexed for 10 s and incubated at 37 °C for 2 h. Absorbance of the supernatant was measured at 600 nm using a spectrophotometer (Perkin Elmer, Lambda 25, USA). The auto-aggregation coefficient (AC) was calculated according to Eq. 1 [10]:

1 $$ {\mathrm{AC}}_{\mathrm{t}}\left(\%\right)=\left[1-\left({\mathrm{OD}}_{2\mathrm{h}}/{\mathrm{OD}}_{\mathrm{i}}\right)\right]\times 100 $$

where, OD_i_ is the initial optical density of the microbial suspension at 600 nm.

For the co-aggregation assay an equal volume (2 mL, 2 × 10^8^ CFU/mL) of *P. acidilactici* Kp10 and pathogenic bacterium (*L. monocytogenes* ATCC 15313) cultures were mixed, vortexed for 10 s and incubated at 37 °C for 2 h. Each control tubes contained 4 mL of each bacterial suspension. The supernatants were measured at OD_600 nm_ and co-aggregation was calculated according to Eq. 2 [11]:

2 $$ \mathrm{Co}\hbox{-} \mathrm{aggregation}\left(\%\right)=\left[1-{\mathrm{OD}}_{\mathrm{mix}}/\left({\mathrm{OD}}_{\mathrm{strain}}+{\mathrm{OD}}_{\mathrm{pathogen}}\right)/2\right]\times 100 $$

where, OD_mix_ is the optical density of the mixture of *P. acidilactici* Kp10 and *L. monocytogenes* at 600 nm, OD_strain_ is the optical density of *P. acidilactici* Kp10 at 600 nm and OD_pathogen_ is the optical density of *L. monocytogenes* at 600 nm. Experiments were conducted in triplicates on two separate occasions.

#### Adhesion of *P. acidilactici* Kp10 cell to solvents

Adhesion of *P. acidilactici* Kp10 cell to solvents was assayed according to the method as described previously [12] with some modifications. Three tubes each containing 3 mL of *P. acidilactici* Kp10 cell (grown in M17 broth at 37 °C for 18 h) suspension in PBS (pH 7.2) at 10^8^ CFU/mL, were each mixed with 1 mL of xylene, chloroform, ethylene acetate and n-hexadecane. The mixture was then vortexed for 1–2 min and allowed to stand for 5–10 min to allow separation of the mixture into two phases. The aqueous phase was measured at 600 nm using a spectrophotometer (Perkin Elmer, Lambda 25, USA). Bacterial affinities to solvents (BATS) with different physicochemical properties (hydrophobicity and electron donor–electron acceptor interactions) were expressed using Eq. 3:

3 $$ \mathrm{BATS}\left(\%\right)=\left(1-{\mathrm{A}}_{10 \min }/{\mathrm{A}}_{0 \min}\right)\times 100 $$

Where, A_10min_ is the absorbance at *t* = 10 min and A_0min_ is the absorbance at *t* = 0 min.

In a separate experiment, Congo red dye method was used to further investigate the cell surface hydrophobicity of *P. acidilactici* Kp10. Agar plates were initially prepared by mixing 2% (*w*/*v*) NaCl in de Man, Rogosa and Sharpe (MRS) medium (Merck, Darmstadt, Germany), followed by the addition of sterile 0.03% (*w*/*v*) Congo red to the mixture. The bacterial strain was then cross-streaked and incubated at 37 °C for 24 h. The colonies stained red were hydrophobic whereas the colorless colonies were considered as non-hydrophobic [13].

#### Survivability studies on tolerance to phenol

Study on the tolerance of *P. acidilactici* Kp10 to phenol was performed by inoculating the cultures in M17 broth with and without phenol. The samples (100 μL) were then spread-plated onto MRS agar and incubated at 37 °C for 24 h. Bacterial survivability was enumerated using the formula as described previously [14].

#### Transmission electron microscopy (TEM) for detection of the S-layer

Cell suspensions of *P. acidilactici* Kp10 and *Lactobacillus crispatus* DSM 20584 (used as a control) were centrifuged at 5000×g for 10 min. The supernatants were pipetted and the pellets fixed in 2.5% glutaraldehyde in 0.1 M sodium cacodylate buffer for 4 to 6 h. The samples were then centrifuged and the supernatants pipetted to remove the fixative. A few drops of horse serum were added to each of the pellets. The coagulated pellets were then diced into 1 mm pieces. Following three washings with sodium cacodylate buffer the samples were post-fixed in 1% aqueous osmium tetroxide and dehydrated in ascending grades of acetone concentrations (30, 50, 75, 80, 95 and 100%). Samples were then infiltrated overnight with an equal mixture (1:1) of resin and acetone. The samples were infiltrated with 100% resin in the following morning and dropped into resin-filled, pre-labeled BEEM capsules and polymerized at 60 °C for 16 h. Ultrathin sections on copper grids were stained with uranyl acetate and lead citrate and examined under the TEM. Cross sections of bacterial cells were examined to detect the S-layer in the cell wall of both strains.

#### Haemolytic activity

The haemolytic activity of *P. acidilactici* Kp10 was determined by growing the bacterial strain in M17 agar at 37 °C for 18 h, and then streaked onto Columbia Agar plates containing 5% *v*/v of sheep blood (BioMeŕieux, Hazelwood, MO, USA). The plates were incubated at 37 °C overnight. Haemolytic reactions were recorded by the presence of a clear zone (*β*-haemolysis), green zone (*α*-haemolysis) or the absence of zone (*γ*-haemolysis) around the colonies [15].

### Determination of starter culture properties

#### Enzymatic characterization

API ZYM strips (API Identification Systems, bioMérieux, France), according to the manufacturer’s instructions, were used to determine the enzymatic characteristics of *P. acidilactici* Kp10. The strips were incubated at 37 °C for 4 h, and the reagents were then added. The color intensity was assessed according to the manufacturer’s color chart. The test was performed in triplicates.

#### Acidification and coagulation activities

Effect of acidification and coagulation activities of *P. acidilactici* Kp10 was assayed by its inoculation into 10% skim milk at 1% level which incubated at 30 °C. The activities were evaluated by observation for commencement of clotting followed by pH measurement after 72 h [16].

#### Qualitative proteolytic activity and starch hydrolysis

*P. acidilactici* Kp10 culture was streaked on M17 agar for 24–48 h. Heavy inoculum of the culture was then streaked on skim milk agar and M17-starch agar and incubated at 37 °C for 24–48 h. Clear zone surrounding colonies on skim milk agar indicated proteolytic activity. To detect the hydrolysis of starch, M17-starch agar was topped with iodine solution [17]. *L. monocytogenes* ATCC 15313 and *E. coli* ATCC 25922 were used as negative controls.

## Results and discussion

The inhibitory activity of the probiotic strain plays an important role in competing with other microorganisms in the gastrointestinal tract (GIT) protecting the latter from being colonized by food-borne pathogens. The inhibitory spectra of *P. acidilactici* Kp10 against different gram-positive and gram-negative bacteria in the present study showed an antagonistic effect of the growth of gram-positive and gram-negative pathogenic microorganisms. The potential probiotic bacterial strain in this study demonstrated an inhibitory activity against *L. monocytogenes* ATCC 15313, *S. enterica* ATCC 13311, *Sh. sonnei* ATCC 9290, *K. oxytoca* ATCC 13182, *E. cloaca* ATCC 35030, *St. pyogenes* ATCC 12378 (Table 1). There was significant difference (*P* < 0.05) between the inhibitory spectrum of *P. acidilactici* Kp10 against *L. monocytogenes* 15313 and five other strains while no significant differences (*P* > 0.05) was observed in inhibitory spectrum of Kp10 against these five strains. To date there are limited reports concerning the inhibitory effects of LAB on gram-negative bacteria due to the structure of their bacterial cell envelopes which is much more complex compared to that of gram-positive bacteria [18]. Their resistance to many antimicrobial agents is attributed to an effective permeable barrier of lipopolysaccharide layer of the outer membrane.

**Table 1:** Inhibitory spectrum of *P. acidilactici* Kp10 against gram-positive and gram-negative bacteria

Microorganism	Zone diameter (mm)
*L. monocytgenes* ATCC 15313	21 ± 0.1^a^
*S. enterica* ATCC 13311	11 ± 0.05^b^
*Sh. sonnei* ATCC 9290	11 ± 0.8^b^
*K. oxytoca* ATCC 13182	11 ± 0.03^b^
*E. cloaca* ATCC 35030	11 ± 0.5^b^
*S. pyogenes* ATCC 12384	11 ± 0.7^b^
*P. acidilactici* Kp10	0

Data are mean values ± SD (*n* = 3)

Values with different superscript letters (a and b) are significantly different (*P* < 0.05)

**Fig. 1:**
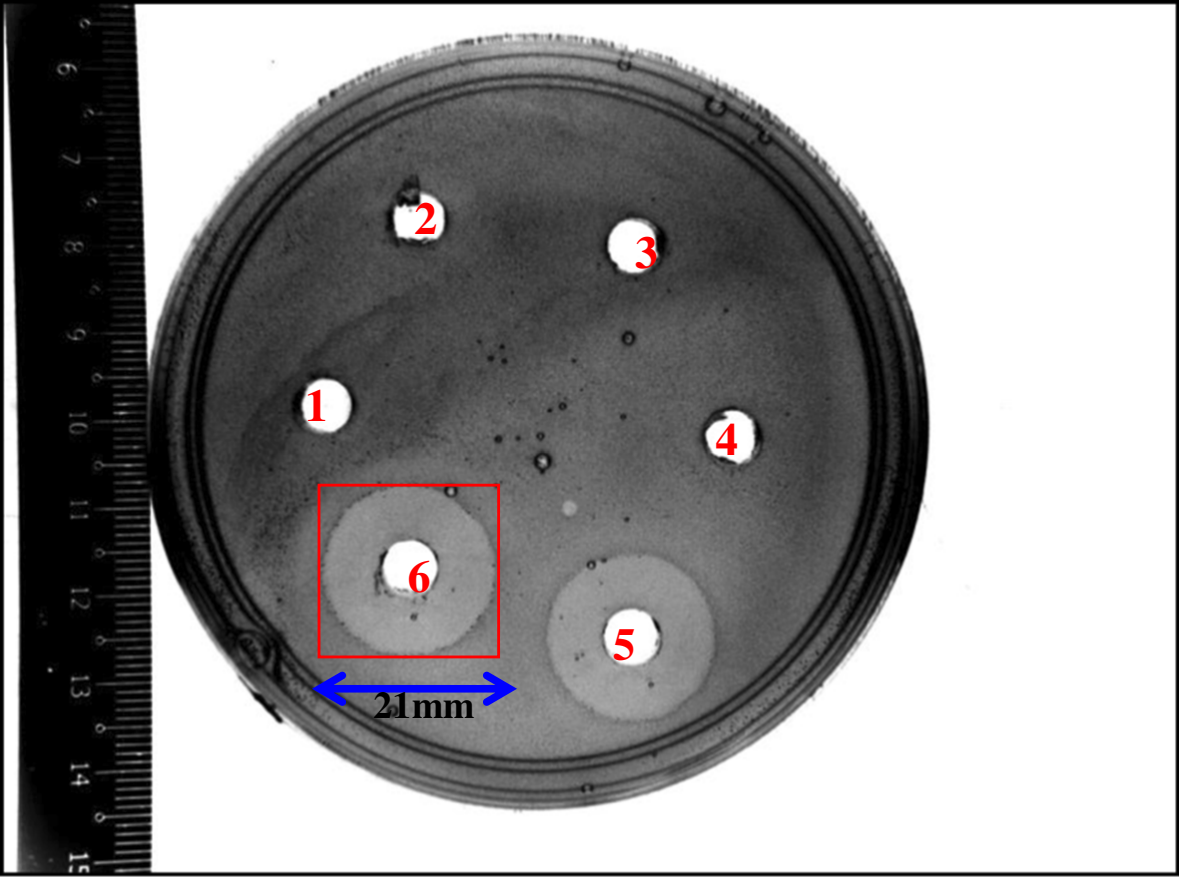
Antimicrobial activity of *P. acidilactici* Kp10 against *L. monocytogenes* ATCC 15313 determined by agar well diffusion method (1 and 2: water; 3 and 4: media; 5 and 6: CFCS of *P. acidilactici* Kp10)

*P. acidilactici*Kp10 inhibited the growth of *L. monocytogenes* which is an important food-borne pathogen (Fig. 1). This observation could infer that *P. acidilactici* Kp10 has the potential to be used as a probiotic microorganism to overcome some major challenges facing the food industry and regulatory agencies. In addition, Kp10 was resistant to its own BLIS as indicated by the absence of activity around the well (Fig. 1). All bacteriocin producing isolates could protect themselves from the adverse effect of their own bacteriocins by the production of an immune protein commonly linked to the C-terminal domain of the bacteriocin [19]. Our finding is in agreement with the earlier reports which stated that bacteriocin producer could protect itself from the adverse effect of its own antimicrobial compounds by a defense system which is expressed concomitantly with the antimicrobial peptide(s) [20, 21]. Some bacteriocinogenic strains have no receptors which would then absorb their own bacteriocins thus rendering the bacteriocin ineffective against their own producer strain. Bacteriocin action and bacteriocin resistance were demonstrated to be contributed by the cell wall as well as its membrane lipid composition. As shown in Fig. 1, two zones of inhibition were observed. During the initial phase of incubation there was high antimicrobial activity which was demonstrated by an inner clear zone. During incubation there was an accompanying increase in pH of the substrate whence the antimicrobial range of activity was approaching its optimum. The antimicrobials further inhibit the growth of the microorganism in the area of the peripheral zone where the concentration of antimicrobials are lower than that presence in the central area [22]. However, it could be result of the presence of more than one bacteriocin.

**Fig. 2:**
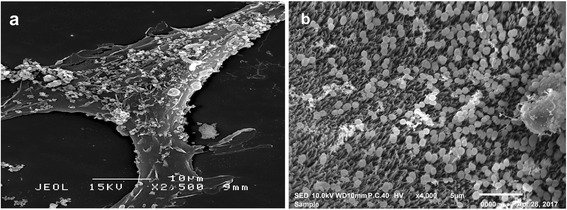
SEM showing adhesion of *P. acidilactici* Kp10 to the surface of: **a** Vero cells, and **b** mucosal epithelium of goat ileum

Adhesion of *P. acidilactici* Kp10 to Vero cells and goat ileum mucosal epithelium as observed under the scanning electron microscope (SEM) are shown in Fig. 2a and b. To our knowledge previous reports on the adhesion of LAB were tested in rats intestine [23], columnar epithelial cells of pigs and calves [8] and ileum of Landrace pigs [10]. The objective of this part of our study was to test qualitatively the colonization of LAB onto epithelial cells. As probiotic could be used in both human and animals we therefore examined LAB colonization in an animal species which have not been previously reported and in this case the goat. Human epithelial cells were not used as these cells were not easily available from our perspective. The goat being a ruminant is thus a species which is most remotely related to the human; however surprisingly our results demonstrated that LAB are capable of colonizing the goat epithelium which further augment our claim that *P. acidilactici* Kp10 is applicable to both human and animals. Colonization with extended transit time is most critical for optimal expression of general and specific physiological functions of probiotic microorganisms. Probiotic strains invariably should demonstrate the ability to adhere to the surface mucosal epithelial cells, an important requirement with reference to effective colonization [24]. Cell adhesion which involve contact between the cell membrane of the bacteria and that of the mucosal epithelium is no doubt a complex process. There were a number of constrains in the evaluation of bacterial adhesion capability in vivo especially in humans. These constrains had prompted a number in vitro studies to be undertaken instead which were directed towards screening bacterial strains with adhering potentials.

For the beneficial effect of probiotics to manifest, there is a need to achieve an adequate mass through aggregation. In a number of ecological niches auto-aggregation, which are cell aggregation between microorganisms of similar strain or co-aggregation, aggregation of genetically different strain, are of considerable importance [25]. LAB with aggregation ability and hydrophobicity cell surface could be more capable to adhere to intestinal epithelial cells. It has been reported that some LAB can prevent adherence of pathogens to intestinal mucosa either by forming a barrier via auto-aggregation or by co-aggregation with the pathogens [26–28]. Invariably cell adherence properties are aggregation ability related.

Auto-aggregation of probiotics appeared to be necessary for the adhesion to intestinal epithelial cells. In addition, the ability to co-aggregate with pathogens may form a barrier which prevents colonization by pathogens. Adherence of bacterial cells is usually related to cell surface characteristics [29, 30]. Hydrophobicity, one of cell surface physicochemical characteristics could affect auto-aggregation and adhesion of bacteria to different surfaces [25]. It was reported that auto-aggregation of LAB is associated with their adhesion ability [28].

The co-aggregation ability could allow LAB strains to inhibit the growth of pathogens in the gastrointestinal and urogenital tracts [31]. Furthermore, LAB strains have a major influence on the micro-environment around the pathogens and in the process of co-aggregation increase the concentration of antimicrobial substances secreted [26, 32]. Additionally, co-aggregation of inhibitor-producing LAB with the pathogens could possibly constitute an important host defense mechanism in the urogenital and GIT. The ability of LAB to co-aggregate with gut pathogens could potentially be a probiotic property of the microorganism [25].

Thus, the potential of *P. acidilactici* Kp10 as a probiotic strain was evaluated for its auto-aggregation and co-aggregation ability with a foodborne pathogenic bacterium, *L. monocytogenes*. *P. acidilactici* Kp10 had higher auto-aggregation values (35.2%) compared to that of *L. monocytogenes* ATCC 15313 (24.7%). *P. acidilactici* Kp10 had a co-aggregation ability with *L. monocytogenes* ATCC 15313 of about 46% (Table 2). Our results concurred with that reported previously [11] for *P. acidilactici* KACC 12307 which had auto-aggregation and co-aggregation values of 35.2 and 46%, respectively. It was also reported that probiotics had higher auto-aggregation abilities than the pathogens [26, 33].

**Table 2:** Aggregation abilities of *P. acidilactici* Kp10 and *L. monocytogenes* ATCC 15313

	*P. acidilactici* Kp10	*L. monocytogenes*ATCC 15313
Auto-aggregation (%)	35.2 ± 0.07^a^	24.7 ± 0.1^b^
	*P. acidilactici* Kp10 with *L. monocytogenes*ATCC 15313	
Co-aggregation (%)	46 ± 0.6	

Mean (± standard deviation) of results from three separate experiments

Values with different superscript letters (a and b) are significantly different (*P* < 0.05)

Cell surface hydrophobicity is another physicochemical property that facilitates first contact between microorganisms and host cells. This non-specific initial interaction is weak and reversible and precedes the subsequent adhesion process mediated by more specific mechanisms involving cell-surface proteins and lipoteichoic acids [34–36]. Thus the contribution of hydrophobicity to adhesion capacity could probably be due to the lack of correlation between hydrophobicity and bacterial adhesion [37–39].

Affinity for chloroform, an acidic and monopolar solvent, reflected the reducing (alkalic) nature of the bacterium. However, its affinity to ethylacetate, an alkalic and monopolar solvent, reflected the oxidizing (acidic) nature of the bacterium. Furthermore, affinity towards apolar solvents (hexadecane and xylene) demonstrated the hydrophobic nature of the bacterium. High hydrophobicity is linked to glycoproteins on the bacterial surface while low hydrophobicity is linked to the presence of polysaccharides on the bacterial surface [40].

**Fig. 3:**
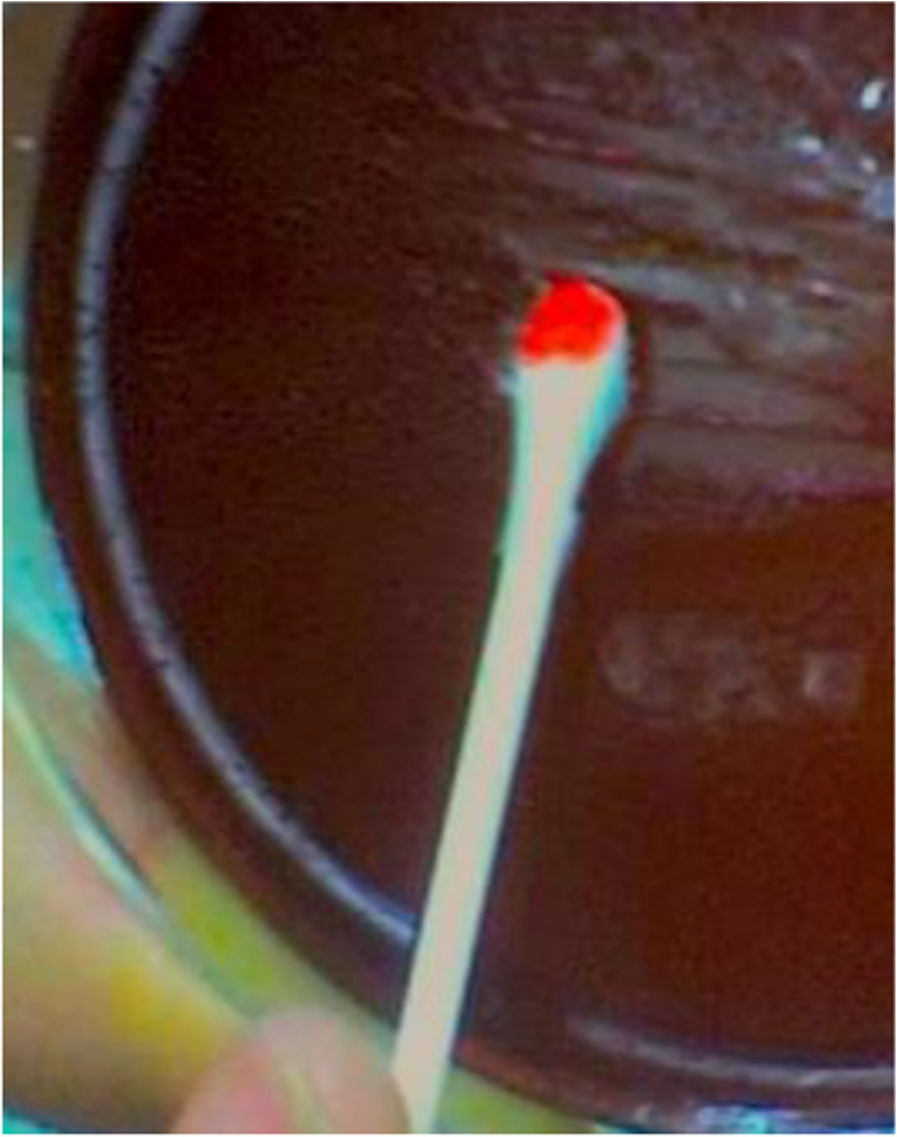
Cell surface hydrophobicity of *P. acidilactici* Kp10 with Congo *red dye*

The adhesion ability of *P. acidilactici* Kp10 to four different solvents (chloroform, xylene, ethylacetate and n- hexadecane) are summarized in Table 3. *P. acidilactici* Kp10 has a strong affinity (46.97%) for xylene, indicating the cells were hydrophobic. The Lewis acid-base characteristics of the cell surface of *P. acidilactici* Kp10 was assessed by its adhesion to chloroform and ethyl acetate. The results showed that *P. acidilactici* Kp10 had a stronger/higher affinity to chloroform (12.42%), an acidic solvent and electron acceptor compared to that of ethyl acetate (5.67%) a basic solvent and electron donor. *P. acidilactici* Kp10 showed a low hydrophobicity (14.55%) for n-hexadecane and positive to Congo red by the presence of red colonies on the agar plate, indicating that it has the hydrophobic structures in its cell wall (Fig. 3).

**Table 3:** Adhesion of *P. acidilactici* Kp10 to xylene, chloroform, ethyl acetate and n- hexadecane

Solvent	Xylene	Chloroform	Ethyl acetate	n- hexadecane
Adhesion (%)	46.97 ± 0.01^a^	12.42 ± 0.01^c^	5.67 ± 0.04^d^	14.55% ± 0.1^b^

Mean (± standard deviation) of results from three separate experiments

Values with different superscript letters (a, b, c, d) are significantly different (*P* < 0.05)

Some aromatic amino acids derived from dietary or endogenously produced proteins that can be deaminated by gut bacteria leading to the formation of phenolic compounds [41]. These compounds exert a bacteriostatic effect against some bacterial strains. The survivability test of probiotics in the intestine refers to their resistance to 0.4% phenol, a catabolic product of aromatic amino acids with bacteriostatic activity [14]. The tolerance of *P. acidilactici* Kp10 to phenol for 24 h is shown in Table 4. Growth of the bacterium was not markedly inhibited as the bacterial strain could still grow in the presence of 0.1% phenol during the incubation. Results showed that *P. acidilactici* Kp10 was moderately tolerant to phenol. A similar result was also reported for *Lb. plantarum* Lp-115 [28]. Bacteria that are tolerant to phenols may have better chances of survival in the GIT. Some LAB strains such as *Lb. acidophilus* DC601, *Lb. gasseri* BO3, *Lb. paracasei* BO52 are tolerant to high phenol concentrations (0.4 to 0.5%) [14, 42], although the physiology of these bacteria are closely related to *P. acidilactici* Kp10.

**Table 4:** Tolerance of *P. acidilactici* Kp10 cells to phenol

M17+ % of phenol	Viable counts^a^ (Log_10_ CFU/ mL)		
	T_0_	T_24_	Inhibition^b^
Blank (without phenol)	5.09 ± 0.01	7.56 ± 0.0	−2.47
0.1	5.04 ± 0.0	6.47 ± 0.0	−1.43
0.2	5.04 ± 0.15	4 .75 ± 0.13	0.29
0.3	5.06 ± 0.06	4.11 ± 0.08	0.95
0.4	5.07 ± 0.25	3.48 ± 0.0	1.59

^a^Log mean counts of three trials (mean ± S.E)

^b^Inhibition = log_10_(initial population) − log_10_(final population)

**Fig. 4:**
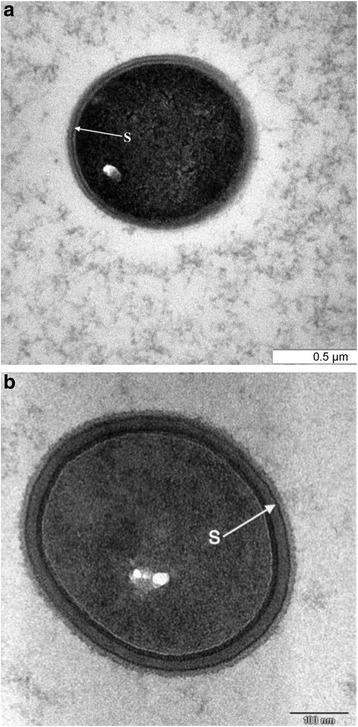
TEM of a cross-section of (**a**) *P. acidilactici* Kp10 and (**b**) *Lb. crispatus* DSM 20584 cells showing the S-layer (*arrow*) in the cell wall of the bacterium

Transmission electron micrographs of the *P. acidilactici* Kp10 and *Lb. crispatus* DSM 20584 (DSM: Deutsche Sammlung von Mikroorganismen un Zellkulturen GmbH/Braunschweig, Germany) are shown in Fig. 4a and b, respectively. From the micrographs, it can be seen that S-layers were presence in the cell wall of both strains. In *P. acidilactici* Kp10, the S-layer was located in the middle of the thick cell wall. However, the S-layer of *Lb. crispatus* DSM 20584 was located more superficially in the bacterial cell wall. S-layer or crystalline surface layer is a common feature of eubacteria and archaebacteria [43]. The structure is composed of identical subunits consisting of a single protein species linked to each other as well as to the supporting cell wall, also known as specific hydrophobic cell surface proteins [44]. The biological functions of the S-layer in eubacteria include protection, cell adhesion and surface recognition [45]. The S-layer protein from *Lb. crispatus* JCM 5810 was also involved in adhesion [46] and the inhibition of adhesion of *E. coli* to the basement membrane of mucosal epithelium [47]. With reference to the function of the S-layer it could be a contributing factor in the adhesion of *P. acidilactici* Kp10 to Vero cells and the intestinal mucosa of goat ileum as observed in the present study. A more conclusive identification of this structure could be obtained by generating an antibody against the specific hydrophobic cell surface protein and gold-labeling the antibody [48].

The absence of pathogenicity traits such as the absence of haemolytic activity in cultures, as observed in this study, suggested the suitability of application of *P. acidilactici* Kp10 in foods [49]. The absence of haemolytic activity is considered a safety prerequisite for the selection of a probiotic strain [50]. *P. acidilactici* Kp10 exhibited γ-haemolytic activity (no haemolysis) when grown in Columbia blood agar. Similar observations were reported in *Lb. paracasei subsp. paracasei*, *Lactobacillus spp.* and *Lb. casei* isolated from dairy products which showed γ-haemolysis except of few that showed α-haemolysis [51]. Most of the LAB strains (69 from 71 strains) have been reported as γ-haemolytic (i.e. no haemolysis) [52].

Application of the commercial API-ZYM is for the selection of strains as potential starter cultures based on superior enzyme profiles especially peptidases and esterases. The test system is also applicable in the determining accelerated maturation and flavor development of fermented products [53]. Esterase in particular from LAB may be involved in the development of fruity flavors and quality improvement in dairy and meat products such as cheese, cured bacon and fermented sausages [54]. Enzymatic activities of *P. acidilactici* Kp10 as evaluated by the semi-quantitative API-ZYM system is shown in Table 5. *P. acidilactici* Kp10 exhibited a very low level of alkaline phosphatase, a lipolytic enzyme. Kp10 demonstrated strong peptidase (leucine-arylamidase) and esterase-lipase (C4 and C8) activities. Proteinases (trypsin) activity is however absent in Kp10. The above are two possible desirable traits for the production of typical flavor. Similar results have been reported on the use of LAB as a starter culture and potential technological implications by increasing desirable flavor in seafood products [55–57].

**Table 5:** Enzyme activities of *P. acidilactici* Kp10

	Enzyme	Production
1	Control	−
2	Alkalinephosphatase	+
3	Esterase (C4)	++
4	Esteraselipase (C8)	++
5	Lipase (C14)	−
6	Leucinearylamidas	++
7	Valinearylamidase	−
8	Cystinearylamidas	−
9	Trypsin	−
10	α-chymotrypsin	++++
11	Acidphosphatase	+
12	Naphthol-AS-BI-phosphohydrolase	++
13	α-galactosidase	++++
14	β-galactosidase	++++
15	β-glucuronidase	−
16	α-glucosidase	++++
17	β-glucosidase	++++
18	N-acetyl-b-glucosaminidase	−
19	α-mannosidase	−
20	α-fucosidase	−

‘+’ refers to positive reaction; ‘-’ refers to negative reaction

Acidification is an important technological and functional property in the selection of LAB as a starter culture [58]. It was found that *P. acidilactici* Kp10 acidified the skim milk used by lowering the pH to 5.3 apart from showing strong coagulating activities. The potential of LAB strains for application as a starter or adjunct cultures in the production of fermented products is demonstrated by their ability to coagulate milk. Results showed that *P. acidilactici* Kp10 exhibited proteolytic activity which is in agreement with the reports published by [59] and [60] for other LAB. LAB are weakly proteolytic compared with other groups of bacteria such as *Bacillus, Proteus, Pseudomonas* and *Coliforms* [61] but the bacterial strains do cause a significant degree of proteolysis in many fermented dairy products [62]. LAB is capable of hydrolyzing oligopeptides into small peptides and amino acids as it possess a very comprehensive proteinase/peptidase system [63]. Many dairy starter cultures are proteolytic thus bioactive peptides can be generated and used in the manufacturing of fermented dairy products. To prepare an experimental starter the technological properties of LAB should include growth, acidifying, proteolytic and amylolytic activities [64].

*P. acidilactici* Kp10 showed positive results for amylolactic activity. Amylases produced by amylolytic LAB (ALAB) facilitate hydrolysis and fermentation of starch to lactic acid in a single step process [65]. ALAB can thus be utilized in commercial production of lactic acid from starchy materials and in reducing the viscosity of starchy complementary foods [66, 67]. Apart from altering the microstructure of starch, ALAB could also modify the amylography and viscosity of starch. α-amylases of ALAB has the ability of partially hydrolyzing raw starch and as such this microorganism could ferment different types of amylaceous raw materials viz. wheat, potato and different starchy substrates [68]. Taking into consideration the global importance and availability of starchy biomass, production of amylases and lactic acid from starch present two potential industrial applications of ALAB. Bulk production of amylases through microbial fermentation could beneficially be utilized in starch degradation which could supply 25–33% of the global enzyme market [69]. Direct conversion of starchy materials to lactic acid by LAB with ability in secreting amylolytic enzymes in a single-step production process is preferred at industrial scale. This approach will eliminate the two-step process, which include enzymatic saccharification for stach hydrolysis followed with LAB fermentation to convert sugar to lactic acid, Production cost could be substantially reduced with a sing-step process to ensure it is economically viable.

## Conclusion

Results from the present study provided ample evidences to claim that *P. acidilactici* Kp10 is a potential probiotic and starter culture. However the data generated were based purely on in vitro studies. In order to claim that this microorganism is categorically a probiotic strain, the survivability and ability to express its probiotic potential in the gastrointestinal environment is also the important criterion to be considered. The environment in the gastrointestinal tract is not only different from that of in vitro*,* there are also a number of the interacting factors that have major influences on the survivability and its probiotic characteristics. The robust environment at industrial scale may not be favourable to the performance and the capability of the selected probiotic strain. To support the recommendation of using *P. acidilactici* Kp10 in food industry, a comprehensive study to identify their comparative advantages is required. However, this is not the objective of this paper. The results obtained from the present in vitro studies gave ample evidences to indicate that *P. acidilactici* Kp10 is a promising probiotic and starter culture potential. However a comprehensive in vivo investigations are required to categorically substantiate its true potential.

## Abbreviations

AC: Auto-aggregation coefficient
ALAB: Amylolytic LAB
BATS: Bacterial cell adhesion to solvent
BLIS: Bacteriocin-like inhibitory substances
DMEM: Dulbecco’s modified eagle medium
GIT: Gastrointestinal tract
LAB: Lactic acid bacteria
MRS: de Man, Rogosa and Sharpe
OD: Optical density
PBS: Phosphate-buffered saline
RPMI: Roswell park memorial institute medium
SEM: Scanning electron microscope
TEM: Transmission electron microscope
